# Health-related quality of life and productivity burden for non-professional caregivers of adults with rare diseases: a real-world study

**DOI:** 10.1186/s13023-025-03796-z

**Published:** 2025-06-06

**Authors:** Ben Johnson, Gregor Gibson, Daniel Baskerville, Giorgio Castellano, Jonathan de Courcy, Halima Iqbal, James Piercy, Angela Williams, Rafael Pinedo-Villanueva, Angela Rylands

**Affiliations:** 1 Kyowa Kirin International, 2 Globeside, Fieldhouse Lane, Marlow, SL7 1HZ UK; 2 Adelphi Real World, Bollington, UK; 3 Nuffield Department of Orthopaedics, Rheumatology and Musculoskeletal Sciences, University of Oxford, Oxford, UK

**Keywords:** Caregiver burden, Productivity, Health-related quality of life, Rare diseases, Real-world

## Abstract

**Background:**

Rare diseases present a substantial patient burden, but the impact on non-professional caregivers is poorly understood. We explored the health-related quality of life (HRQoL) and productivity burden on caregivers of adults with rare diseases.

**Methods:**

We analysed physician- and caregiver-reported real-world data from France, Germany, Italy, Spain, the United Kingdom, and the United States of America collected July 2017–March 2021 via Adelphi Disease Specific Programmes™ in amyotrophic lateral sclerosis (ALS), eosinophilic esophagitis (EoE), graft versus host disease (GvHD), Huntington’s disease (HD), myasthenia gravis (MG), and progressive supranuclear palsy (PSP). Non-professional caregivers completed the EQ-5D-5L and Work Productivity and Activity Impairment questionnaire. Multivariate regression analysis modelled the relationship of care recipient/caregiver characteristics with caregiver HRQoL and productivity.

**Results:**

Data were provided by 365 caregivers; 114, 89, 75, 32, 29 and 26 in GvHD, PSP, ALS, MG, EoE and HD, respectively. Care recipients’ mean (standard deviation [SD]) age was 58.7 (15.6) years, 59% were male and 23% had both professional and non-professional caregivers. Patients’ mean (SD) EuroQol visual analogue scale (EQ VAS) score was 50.9 (23.3) and mean EQ-5D utility was 0.460 (0.350). Caregivers’ mean age was 55.8 (13.8) years, 66% were female. Caregivers’ EQ-5D-5L indicated their greatest problems in anxiety/depression. Overall, 45% of caregivers were employed, mostly part-time. In the past 7 days, mean (SD) caregiver absenteeism was 5.2% (13.1%), presenteeism was 28.0% (23.7%), and activity impairment was 43.1% (27.2%). Regressions identified multiple significant associations with caregivers’ HRQoL and productivity. Caregivers’ HRQoL (EQ-5D utility and EQ VAS) was associated with care recipients’ EQ-5D utility and caregivers’ age. Outcomes relating to caregivers’ employment and productivity (hours spent caring, employment status, hours in employment, hours of employment missed, absenteeism, presenteeism, work impairment and activity impairment) were most frequently associated with care recipients’ EQ-5D utility, caregivers’ age and sex, caregiver living with the care recipient, the presence of a professional caregiver, and the care recipient having HD.

**Conclusions:**

The substantial burden of providing non-professional caregiving to adults with rare diseases is associated with multiple factors. Interventions improving care recipient HRQoL could enhance caregiver HRQoL and productivity.

**Supplementary Information:**

The online version contains supplementary material available at 10.1186/s13023-025-03796-z.

## Background

A rare disease is a medical condition affecting a small proportion of the population [1–3]. They are often chronic, progressive, genetic, inherited conditions that can be life-shortening, and that may have no cure [3, 4]. Clinical guidelines and standard of care are often lacking in rare diseases [5]. The lack of effective treatments leads to polypharmacy [6] and extensive use of drugs “off label” [7].

The threshold to consider a medical condition to be a rare disease depends on geographical location, with international prevalence thresholds ranging from 5 to 76 per 100,000 [8–12]. European orphan drugs regulations specify that for a condition to be classified as a rare disease, its prevalence must be no greater than 50 per 100,000 [8]. The US Rare Diseases Act defined any disorder affecting fewer than 200,000 US citizens as a rare disease [13], a definition used in FDA’s 2019 Draft Guidance for Industry “Rare Diseases: Common Issues in Drug Development” [14]. Based on Department of Commerce figures, this equated to a prevalence of approximately 60 per 100,000 in the United States of America (USA) at the start of 2022 [15].

Although the prevalence of individual rare diseases is relatively low, given the number of rare diseases, together they affect a substantial number of people. The Orphanet database holds data on 6172 different rare diseases [4], a recent international review suggested there are more than 10,000 rare diseases [16], and 6–8000 medical conditions are classified as rare diseases by the European Commission [17]. Based on prevalence estimates and global population numbers, more than 470 million people worldwide were estimated to have rare diseases in 2022 [3]. The European Commission has reported that approximately 30 million people in the EU have rare diseases [17], and the Genetic and Rare Diseases Information Center has reported that 25–30 million people in the USA have rare diseases [18].

The humanistic and productivity burden of rare diseases is considerable; the debilitating symptoms, chronic nature, and lack of effective treatments impact the health-related quality of life (HRQoL) and functional ability of patients [19, 20]. Additionally, patients with rare diseases frequently require the support of one or more caregivers, often family members. Impaired HRQoL is also reported in non-professional caregivers of patients with rare diseases [20–22].

The total cost of rare diseases in the US in 2019 was estimated at $997 billion [23], with this cost including $437 billion in indirect costs attributed to productivity losses for patients and their non-professional caregivers. A study to assess the costs of ten different rare diseases across eight European countries concluded that average annual costs per patient related to lost productivity could be as high as €14,444, although this varied markedly across the participating countries [24].

A number of published studies show a substantial burden for caregivers of patients with rare diseases, indicating a growing awareness of the importance of this topic. However, many studies consider a single disease within one country [25–30]. Such studies frequently focus on parents as caregivers, and few investigate the specific drivers of caregiver burden [26–33]. The Social Economic Burden and Health-Related Quality of Life in Patients with Rare Diseases in Europe (BURQOL-RD) project aimed to quantify the socio-economic burden and HRQoL impact of ten rare diseases for patients and their caregivers in Europe [21, 34]. Data from this study demonstrate that the type of disease and the health/social care systems of the country of residence impact the burden of caregiving in rare diseases [24].

The objective of the current study was to explore the HRQoL and productivity impact of caring for adults with a rare disease, and to investigate the association of care recipient and caregiver characteristics with the HRQoL and productivity of the caregiver, independently of the type of rare disease or country.

## Materials and methods

### Study design and data source

This was a retrospective analysis of physician- and caregiver-reported data drawn from six Adelphi Disease Specific Programmes (DSP™) conducted between July 2017 and March 2021 in five European countries and the USA. DSPs are large, multinational, cross-sectional (with retrospective data collection) surveys conducted in real-world clinical practice. Full details of the DSP methodology have been published previously [35].

Specifically, for this study, data were collected from physicians and non-professional caregivers attending a physician visit with a patient. Diseases were selected to provide variety in patient characteristics (such as usual age of onset), disease severity and manifestation (neurological and non-neurological diseases), and caregiver burden, to allow identification of common themes across a broad range of conditions. Diseases were also selected on the pragmatic basis of DSPs providing sufficiently large datasets with data collected in the past five years. Data were from the Huntington’s disease (HD) DSP, collected July 2017 to October 2017; the progressive supranuclear palsy (PSP) DSP, collected August 2018 to October 2018; the graft versus host disease (GvHD) DSP, collected September 2019 to February 2020; the myasthenia gravis (MG) DSP, collected March 2020 to September 2020; the eosinophilic esophagitis (EoE) DSP, collected July 2020 to December 2020; and the amyotrophic lateral sclerosis (ALS) DSP, collected July 2020 to March 2021. An overview of included diseases is provided in Online Appendix Table S1.

Data were collected in France, Germany, Italy, Spain, the UK, and the USA for all DSPs except HD, for which data were not collected in Spain.

### Study participants and eligibility criteria

Physicians were eligible to participate in a DSP if they were personally responsible for the management of people living with one of the diseases under study (care recipients). For data relating to a care recipient to be eligible for inclusion in this analysis, they were required to be at least 18 years old, have a physician-confirmed diagnosis of the relevant disease, and be accompanied on their visit to the physician by a non-professional caregiver who was 18 years or older.

### Study procedures

Physicians completed a patient record form for consecutively visiting patients attending routine care appointments. Completion of the form was undertaken by consulting existing patient clinical records, and according to physician judgement and diagnostic expertise. Caregivers accompanying care recipients were invited to complete a form. The data reported by the caregivers were matched anonymously to the physician’s forms. Physicians, patients and caregivers all provided informed consent to participate.

### Data collected

#### Physician-reported data

The physician-reported patient record form included the age and sex of the care recipient, whether the disease was inherited or there was a known family history, current disease severity, relationship of the caregiver to the care recipient, and the length of time that the care recipient had required a caregiver.

#### Caregiver-reported data

Caregivers reported demographic details for the care recipient and themselves, information regarding caregiving needs, their relationship to the care recipient, whether they lived with the care recipient, time spent caring, details of their employment status, and the impact of caregiving on their paid work.

Caregivers also completed two standardised questionnaires:

The EQ-5D-5L assesses HRQoL and was completed as a proxy for the care recipient and a caregiver self-report. The EQ-5D-5L assesses health status in five dimensions (mobility, self-care, usual activities, pain/discomfort, and anxiety/depression), via five response levels (no problems, slight problems, moderate problems, severe problems, extreme problems/unable to do) [36]. Responses were mapped to EQ-5D-3L outcomes and valued using a UK scoring algorithm [37, 38], resulting in a single EQ-5D utility, with a value of 1 indicating perfect health, a value of 0 indicating death, and negative values suggesting health states worse than death [39]. Mapping from the EQ-5D-5L to the EQ-5D-3L allowed comparison with previously published utilities based on the EQ-5D-3L, and EQ-5D-3L is preferred in economic modelling by decision-makers such as the National Institute for Health and Care Excellence [40]. The EQ-5D-5L also includes a 20 cm visual analogue scale (VAS) on which the respondent rates perceived health from 0 (the worst imaginable health) to 100 (the best imaginable health).

The Work Productivity and Activity Impairment (WPAI) questionnaire assesses productivity and activity during the past 7 days [41], and was completed as a caregiver self-report. The WPAI included six questions concerning employment status, hours worked, hours of work missed due to caregiving, hours of work missed for other reasons, and the degree to which caregiving responsibilities affected productivity at work and regular daily activities (both on a scale of 0 = no effect to 10 = completely prevented). Absenteeism (calculated as: hours of work missed due to caregiving/(hours worked + hours of work missed for any reason) × 100)), presenteeism (calculated as: degree to which caregiving responsibilities affected productivity at work [scale 0 (no effect) to 10 (unable to work)] × 10), overall work impairment (calculated as: sum of absenteeism and presenteeism), and total activity impairment (calculated as: degree to which caregiving responsibilities affected regular daily activities [scale 0 (no effect) to 10 (unable to do daily activities)] × 10) were reported as percentages.

### Statistical analyses

All variables were assessed descriptively: care recipient and caregiver demographic data, current disease severity, whether care recipient received professional care, the relationship of the caregiver to the care recipient, whether the caregiver lived with the care recipient, caregiver employment status and the impact of caregiving on caregivers’ paid work. For continuous variables, sample size, mean, standard deviation (SD) and range were presented, and for categorical variables, sample size and the number and percent in each category were presented.

Multivariate regression analysis was used to model the relationship between caregiver and care recipient characteristics and caregiver HRQoL and productivity. A separate model was run for each of the following dependent variables: time spent caring (hours/week), caregiver currently employed (yes/no), caregiver income reduction (%), hours worked (from WPAI), hours missed in paid employment (from WPAI), absenteeism (%, from WPAI), presenteeism (%, from WPAI), overall work impairment (%, from WPAI), activity impairment (%, from WPAI), caregivers’ self-reported EQ VAS score and caregivers’ self-reported EQ-5D utility. The following were considered for inclusion as independent variables in each model: care recipient proxy-reported EQ-5D utility, care recipient disease (included as a separate binary variable for each disease, with EoE as the reference disease), care recipient age, care recipient sex, caregiver age, caregiver sex, whether caregiver lived with care recipient, whether care recipient received professional care. A number of potential independent variables were excluded from some models due to collinearity with other variables. We used variance inflation factors (VIF) and the Condition Number to detect multicollinearity, with variables excluded if VIF > 10 or condition number > 30. The variable ‘length of care’ was excluded as it caused the base size to drop by > 50% in all models. The proportion of missing data for independent variables included in the regression models was generally low; less than 3% in all cases, with the exception of the variable indicating whether the caregiver lived with the care recipient and the variable indicating whether professional care was provided, which had a higher proportion of missing data at 11% and 10%, respectively.

Logistic regression was used to assess the relationship between independent variables and caregivers currently employed, as the dependent variable is binary in this case. All other regressions were based on linear models as all other dependent variables were continuous. It was verified that all models met the requirements for linear regression (linearity, normality, homoscedasticity and independence) [42]. Independent variables included in regression analysis were considered statistically significantly associated with the dependent variable if p-values were below 0.05.

All analyses were conducted in Stata v17.0 [43].

## Results

### Care recipient characteristics and care requirements

A total of 177 physicians and 365 caregivers provided data for 365 care recipients (Table 1).

**Table 1:** Care recipient characteristics and care requirements

	**Total** **(N = 365)**	**ALS** **(N = 75)**	**EoE** **(N = 29)**	**GvHD** **(N = 114)**	**HD** **(N = 26)**	**MG** **(N = 32)**	**PSP** **(N = 89)**
*Country, n (%)*							
n	365	75	29	114	26	32	89
Germany	88 (24%)	5 (7%)	0 (0%)	51 (45%)	2 (8%)	1 (3%)	29 (33%)
France	81 (22%)	23 (31%)	7 (24%)	30 (26%)	6 (23%)	5 (16%)	10 (11%)
USA	65 (18%)	20 (27%)	8 (28%)	9 (8%)	6 (23%)	9 (28%)	13 (15%)
Spain	66 (18%)	14 (19%)	6 (21%)	16 (14%)	0 (0%)	13 (41%)	17 (19%)
Italy	56 (15%)	10 (13%)	5 (17%)	8 (7%)	10 (38%)	4 (13%)	19 (21%)
UK	9 (2%)	3 (4%)	3 (10%)	0 (0%)	2 (8%)	0 (0%)	1 (1%)
*Inherited/Known family history*^*a*^*, n (%)*							
n	365	75	29	114	26	32	89
Yes	25 (7%)	6 (8%)	0 (0%)	0 (0%)	14 (54%)	0 (0%)	5 (6%)
No	335 (92%)	69 (92%)	29 (100%)	114 (100%)	12 (46%)	32 (100%)	79 (89%)
Don’t know	5 (1%)	0 (0%)	0 (0%)	0 (0%)	0 (0%)	0 (0%)	5 (6%)
*Age, years*							
n	365	75	29	114	26	32	89
Mean (SD)	58.7 (15.6)	63.1 (12.4)	41.4 (17.3)	52.9 (14.0)	50.1 (13.4)	58.5 (17.3)	70.8 (7.0)
Median	62.0	63.0	44.0	54.0	47.5	61.0	71.0
Range	18–90	22–90	18–74	20–90	30–79	24–83	52–87
*Sex, n (%)*							
n	365	75	29	114	26	32	89
Male	216 (59%)	48 (64%)	16 (55%)	70 (61%)	17 (65%)	14 (44%)	51 (57%)
Female	149 (41%)	27 (36%)	13 (45%)	44 (39%)	9 (35%)	18 (56%)	38 (43%)
*Current severity*^*a*^*, n (%)*							
n	328	75	29	77	26	32	89
Mild	118 (36%)	14 (19%)	17 (59%)	47 (61%)	13 (50%)	16 (50%)	11 (12%)
Moderate	134 (41%)	31 (41%)	9 (31%)	22 (29%)	11 (42%)	12 (38%)	49 (55%)
Severe/Very severe	76 (23%)	30 (40%)	3 (10%)	8 (10%)	2 (8%)	4 (13%)	29 (33%)
*Receiving professional caregiving*^*a*^*, n (%)*							
n	330	75	0^b^	110	26	32	87
Yes	75 (23%)	24 (32%)	-	18 (16%)	6 (23%)	4 (13%)	23 (26%)
No	255 (77%)	51 (68%)	-	92 (84%)	20 (77%)	28 (88%)	64 (74%)
*Time caregiver has been required, months*^*a*^							
n	128	57	0^b^	0^b^	0^b^	0^b^	71
Mean (SD)	18.8 (17.4)	11.1 (13.1)	-	-	-	-	24.9 (18.1)
Median	12.0	6.0	-	-	-	-	24.0
Range	0.2–96	1.0–60.0	-	-	-	-	0.2–96
*Time/week caring for care recipient, hours*							
n	306	75	21	93	0	31	86
Mean (SD)	27.2 (30.2)	25.6 (6.0)	19.0 (35.1)	16.8 (15.0)	-	29.3 (35.1)	41.0 (44.0)
Median	24.0	24.0	10.0	12.0	-	15.0	26.5
Range	0–168	24–48	0–168	0–100	-	2–168	0–168

ALS, amyotrophic lateral sclerosis; EoE, eosinophilic esophagitis; GvHD, graft versus host disease; HD, Huntington’s disease; MG, myasthenia gravis; PSP, progressive supranuclear palsy; SD, standard deviation

^a^Physician-reported; ^b^Data missing as not asked

The largest data set in the analysis was GvHD (31% of care recipients). PSP and ALS care recipients together comprised 45% of the study population, with EoE, HD and MG each comprising less than 10% of the study population. Overall, 82% of care recipients were from Europe, with the remainder coming from the USA. Only 2% of patients were from the UK, with at least 15% from each of the other countries. The number of care recipients with each of the six diseases varied across countries: 45% of GvHD care recipients were from Germany, but none from the UK, and 38% of HD care recipients were from Italy, but none from Spain.

Care recipients’ mean (SD) age was 58.7 (15.6) years, with the oldest being those with PSP (mean 70.8 [7.0] years) and the youngest those with EoE (mean 41.4 [17.3] years). Males comprised 59% of care recipients, although this varied between 44% for MG and 65% for HD. The majority of care recipients had no known family history of the relevant disease. Overall, 77% of care recipients were considered by their physicians to currently have mild or moderate disease. The proportions of care recipients reported to have severe disease were highest in ALS (40%) and PSP (33%).

Overall, 23% of patients were receiving professional care alongside their non-professional care. The length of time for which caregiving (professional or non-professional) had been required was reported only for ALS and PSP, and varied from less than a week to 8 years. Reported time spent caring for care recipients each week was wide ranging, with some caregivers reporting providing care for 168 h/week (24 h every day). The highest mean number of hours/week spent caring was reported for care recipients with PSP (41.0 [44.0]), and the lowest for GvHD (16.8 [15.0]).

The most common activities requiring caregiver support were preparing meals/cooking, walking unaided and travelling out of home (data not shown).

### Care recipient health-related quality of life

Care recipient EQ-5D-5L findings are shown in Table 2.

**Table 2:** Proxy-reported care recipient health-related quality of life assessed with the EQ-5D-5L

	Total (N = 365)	ALS (N = 75)	EoE (N = 29)	GvHD (N = 114)	HD (N = 26)	MG (N = 32)	PSP (N = 89)
*Mobility, n (%)*							
n	361	74	29	113	26	31	88
No problems	101 (28%)	7 (9%)	25 (86%)	56 (50%)	6 (23%)	3 (10%)	4 (5%)
Slight problems	60 (17%)	11 (15%)	0 (0%)	26 (23%)	6 (23%)	11 (35%)	6 (7%)
Moderate problems	102 (28%)	23 (31%)	3 (10%)	24 (21%)	10 (38%)	9 (29%)	33 (38%)
Severe problems	61 (17%)	15 (20%)	1 (3%)	6 (5%)	3 (12%)	8 (26%)	28 (32%)
Unable to walk about	37 (10%)	18 (24%)	0 (0%)	1 (1%)	1 (4%)	0 (0%)	17 (19%)
*Self-care, n (%)*							
n	359	74	29	112	26	31	87
No problems	114 (32%)	7 (9%)	27 (93%)	62 (55%)	8 (31%)	3 (10%)	7 (8%)
Slight problems	80 (22%)	16 (22%)	0 (0%)	32 (29%)	11 (42%)	16 (52%)	5 (6%)
Moderate problems	82 (23%)	24 (32%)	1 (3%)	11 (10%)	4 (15%)	7 (23%)	35 (40%)
Severe problems	44 (12%)	12 (16%)	1 (3%)	5 (4%)	0 (0%)	4 (13%)	22 (25%)
Unable to wash or dress	39 (11%)	15 (20%)	0 (0%)	2 (2%)	3 (12%)	1 (3%)	18 (21%)
*Usual activities, n (%)*							
n	361	74	29	113	26	31	88
No problems	71 (20%)	4 (5%)	23 (79%)	35 (31%)	5 (19%)	2 (6%)	2 (2%)
Slight problems	82 (23%)	13 (18%)	0 (0%)	36 (32%)	12 (46%)	13 (42%)	8 (9%)
Moderate problems	112 (31%)	22 (30%)	5 (17%)	30 (27%)	5 (19%)	12 (39%)	38 (43%)
Severe problems	54 (15%	19 (26%)	1 (3%)	10 (9%)	0 (0%)	3 (10%)	21 (24%)
Unable to do usual activities	42 (12%)	16 (22%)	0 (0%)	2 (2%)	4 (15%)	1 (3%)	19 (22%)
*Pain/discomfort, n (%)*							
n	360	74	29	112	26	31	88
No pain/discomfort	84 (23%)	17 (23%)	20 (69%)	20 (18%)	13 (50%)	2 (6%)	12 (14%)
Slight pain/discomfort	114 (32%)	26 (35%)	0 (0%)	43 (38%)	6 (23%)	13 (42%)	26 (30%)
Moderate pain/discomfort	129 (36%)	23 (31%)	8 (28%)	38 (34%)	7 (27%)	13 (42%)	40 (45%)
Severe pain/discomfort	27 (8%)	6 (8%)	1 (3%)	10 (9%)	0 (0%)	3 (10%)	7 (8%)
Extreme pain/discomfort	6 (2%)	2 (3%)	0 (0%)	1 (1%)	0 (0%)	0 (0%)	3 (3%)
*Anxiety/depression, n (%)*							
n	359	73	29	112	26	31	88
Not anxious/depressed	63 (18%)	4 (5%)	15 (52%)	33 (29%)	2 (8%)	4 (13%)	5 (6%)
Slightly anxious/depressed	109 (30%)	15 (21%)	0 (0%)	45 (40%)	9 (35%)	12 (39%)	28 (32%)
Moderately anxious/depressed	124 (35%)	33 (45%)	9 (31%)	24 (21%)	12 (46%)	7 (23%)	39 (44%)
Severely anxious/depressed	44 (12%)	16 (22%)	1 (3%)	5 (4%)	1 (4%)	8 (26%)	13 (15%)
Extremely anxious/depressed	19 (5%)	5 (7%)	4 (14%)	5 (4%)	2 (8%)	0 (0%)	3 (3%)
*EQ VAS score*							
n	356	70	27	114	26	30	89
Mean (SD)	50.9 (23.3)	37.5 (21.4)	82.3 (17.7)	58.0 (19.7)	54.8 (20.1)	54.3 (19.3)	40.5 (19.3)
Median	50.0	40.0	88.0	60.0	57.5	60.0	40.0
Range	0–100	0–95	35–100	0–95	0–90	20–85	0–80
*EQ-5D utility*							
n	357	73	29	111	26	31	87
Mean (SD)	0.460 (0.350)	0.276 (0.342)	0.755 (0.311)	0.624 (0.268)	0.552 (0.296)	0.465 (0.247)	0.277 (0.332)
Median	0.531	0.376	0.925	0.651	0.614	0.560	0.429
Range	− 0.532–0.989	− 0.532–0.891	− 0.091–0.987	− 0.455–0.989	− 0.237–0.871	− 0.036–0.810	− 0.532–0.986

© 2009 EuroQol Research Foundation. EQ-5D™ is a trade mark of the EuroQol Research Foundation

As assessed by proxy completion of the EQ-5D-5L by the caregiver on behalf of the care recipient

ALS, amyotrophic lateral sclerosis; EoE, eosinophilic esophagitis; GvHD, graft versus host disease; HD, Huntington’s disease; MG, myasthenia gravis; PSP, progressive supranuclear palsy; SD, standard deviation; VAS, visual analogue scale

Some degree of difficulty for the EQ-5D-5L dimensions mobility, self-care, usual activities, pain/discomfort, and anxiety/depression was reported for 72%, 68%, 80%, 77% and 82% of patients, respectively. When considering responses by disease, the highest prevalence of problems with mobility, self-care, and usual activities was reported in care recipients with ALS and PSP, the highest prevalence of problems in pain/discomfort was for those with ALS, and the highest prevalence of problems in anxiety/depression was for those with ALS, HD, and PSP.

Both EQ VAS scores and EQ-5D utilities showed very large ranges, indicating considerable variability in care recipient HRQoL. For some care recipients, an EQ VAS score of 0 (‘the worst health I can imagine’) was reported, while for some a score of 100 (the ‘best health I can imagine’) was indicated. For some care recipients, an EQ-5D utility below zero (i.e. a health state worse than death) was estimated, while for others an EQ-5D utility close to 1 (i.e. full health) was obtained. Both EQ VAS scores and EQ-5D utilities indicated that HRQoL was poorest for care recipients with ALS or PSP, with the best HRQoL reported for care recipients with EoE.

### Caregiver characteristics

Caregiver characteristics are reported in Table 3.

**Table 3:** Caregiver characteristics

	Total (N = 365)	ALS (N = 75)	EoE (N = 29)	GvHD (N = 114)	HD (N = 26)	MG (N = 32)	PSP (N = 89)
*Age, years*							
n	360	73	28	113	25	32	89
Mean (SD)	55.8 (13.8)	59.8 (12.5)	48.2 (14.3)	52.0 (12.5)	50.6 (12.5)	55.0 (15.1)	61.5 (13.3)
Median	56.0	60.0	47.0	53.0	45.0	54.5	65.0
Range	20–88	24–85	23–77	20–75	30–75	30–88	27–83
*Sex, n (%)*							
n	362	75	29	114	23	32	89
Male	123 (34%)	28 (37%)	13 (45%)	32 (28%)	6 (26%)	13 (41%)	31 (35%)
Female	239 (66%)	47 (63%)	16 (55%)	82 (72%)	17 (74%)	19 (59%)	58 (65%)
*Relationship to care recipient, n (%)*							
n	363	74	28	114	26	32	89
Partner/Spouse	265 (73%)	59 (80%)	16 (57%)	85 (75%)	20 (77%)	21 (66%)	64 (72%)
Parent/Guardian		0 (0%)	7 (25%)	0 (0%)	0 (0%)	0 (0%)	0 (0%)
Child	7 (2%)	7 (9%)	1 (4%)	8 (7%)	1 (4%)	4 (13%)	21 (24%)
Sibling	42 (12%)	6 (8%)	3 (11%)	5 (4%)	1 (4%)	2 (6%)	3 (3%)
Other relationship^a^	20 (6%)	2 (3%)	1 (4%)	13 (11%)	3 (12%)	2 (6%)	0 (0%)
Other family member	21 (6%) 13 (4%)	0 (0%)	0 (0%)	8 (7%)	1 (4%)	3 (9%)	1 (1%)
*Caregiver lives with care recipient, n (%)*							
n	326	75	0	109	22	32	88
Yes	276 (85%)	63 (84%)	-	95 (87%)	22 (100%)	25 (78%)	71 (81%)
No	50 (15%)	12 (16%)	-	14 (13%)	0 (0%)	7 (22%)	17 (19%)

ALS, amyotrophic lateral sclerosis; EoE, eosinophilic esophagitis; GvHD, graft versus host disease; HD, Huntington’s disease; MG, myasthenia gravis; PSP, progressive supranuclear palsy; SD, standard deviation

^**a**^E.g. friend, neighbour, voluntary carer

Caregivers’ mean ages ranged from 48.2 (14.3) years for those caring for adults with EoE to 61.5 (13.3) years for those caring for adults with PSP, with an overall mean age of 55.8 (13.8) years. In total, 66% of caregivers were female, with a range of 55–74% for carers of individuals with EoE and HD, respectively.

The most common relationship of caregiver to care recipient was partner or spouse. The vast majority of caregivers for whom living circumstances were reported lived with the care recipient, regardless of disease.

### Caregiver health-related quality of life

Caregiver EQ-5D-5L findings are shown in Table 4.

**Table 4:** Caregiver health-related quality of life assessed with the EQ-5D-5L

	Total (N = 365)	ALS (N = 75)	EoE (N = 29)	GvHD (N = 114)	HD (N = 26)	MG (N = 32)	PSP (N = 89)
*Mobility, n (%)*							
n	363	75	29	114	25	32	88
No problems	298 (82%)	58 (77%)	27 (93%)	103 (90%)	22 (88%)	24 (75%)	64 (73%)
Slight problems	39 (11%)	7 (9%)	0 (0%)	9 (8%)	3 (12%)	7 (22%)	13 (15%)
Moderate problems	17 (5%)	5 (7%)	1 (3%)	2 (2%)	0 (0%)	1 (3%)	8 (9%)
Severe problems	6 (2%)	3 (4%)	1 (3%)	0 (0%)	0 (0%)	0 (0%)	2 (2%)
Unable to walk about	3 (1%)	2 (3%)	0 (0%)	0 (0%)	0 (0%)	0 (0%)	1 (1%)
*Self-care, n (%)*							
n	363	75	29	114	25	32	89
No problems	331 (91%)	67 (89%)	28 (97%)	110 (96%)	24 (96%)	26 (81%)	76 (86%)
Slight problems	14 (4%)	0 (0%)	0 (0%)	4 (4%)	1 (4%)	5 (16%)	4 (5%)
Moderate problems	11 (3%)	4 (5%)	0 (0%)	0 (0%)	0 (0%)	1 (3%)	6 (7%)
Severe problems	3 (1%)	2 (3%)	1 (3%)	0 (0%)	0 (0%)	0 (0%)	0 (0%)
Unable to wash or dress	4 (1%)	2 (3%)	0 (0%)	0 (0%)	0 (0%)	0 (0%)	2 (2%)
*Usual activities, n (%)*							
n	364	75	29	114	25	32	89
No problems	301 (83%)	60 (80%)	26 (90%)	99 (87%)	22 (88%)	25 (78%)	69 (78%)
Slight problems	41 (11%)	8 (11%)	0 (0%)	12 (11%)	3 (12%)	6 (19%)	12 (13%)
Moderate problems	15 (4%)	4 (5%)	2 (7%)	3 (3%)	0 (0%)	1 (3%)	5 (6%)
Severe problems	4 (1%)	2 (3%)	1 (3%)	0 (0%)	0 (0%)	0 (0%)	1 (1%)
Unable to do usual activities	3 (1%)	1 (1%)	0 (0%)	0 (0%)	0 (0%)	0 (0%)	2 (2%)
*Pain/discomfort, n (%)*							
n	361	75	29	112	25	32	88
No pain/discomfort	234 (65%)	46 (61%)	26 (90%)	77 (69%)	17 (68%)	20 (63%)	48 (55%)
Slight pain/discomfort	92 (25%)	18 (24%)	0 (0%)	28 (25%)	7 (28%)	9 (28%)	30 (34%)
Moderate pain/discomfort	28 (8%)	6 (8%)	2 (7%)	7 (6%)	1 (4%)	3 (9%)	9 (10%)
Severe pain/discomfort	7 (2%)	5 (7%)	1 (3%)	0 (0%)	0 (0%)	0 (0%)	1 (1%)
Extreme pain/discomfort	0 (0%)	0 (0%)	0 (0%)	0 (0%)	0 (0%)	0 (0%)	0 (0%)
*Anxiety/depression, n (%)*							
n	362	75	29	113	25	32	88
Not anxious/depressed	174 (48%)	30 (40%)	22 (76%)	62 (55%)	13 (52%)	12 (38%)	35 (40%)
Slightly anxious/depressed	119 (33%)	30 (40%)	0 (0%)	33 (29%)	9 (36%)	13 (41%)	34 (39%)
Moderately anxious/depressed	51 (14%)	10 (13%)	6 (21%)	13 (12%)	3 (12%)	5 (16%)	14 (16%)
Severely anxious/depressed	16 (4%)	3 (4%)	1 (3%)	5 (4%)	0 (0%)	2 (6%)	5 (6%)
Extremely anxious/depressed	2 (1%)	2 (3%)	0 (0%)	0 (0%)	0 (0%)	0 (0%)	0 (0%)
*EQ VAS score*							
n	350	70	28	114	25	26	87
Mean (SD)	81.6 (16.2)	79.3 (19.8)	90.9 (12.0)	84.6 (10.3)	80.9 (12.3)	82.7 (15.7)	76.3 (19.4)
Median	85.0	90.0	95.0	85.0	81.0	85.0	80.0
Range	7–100	7–100	47–100	55–100	50–100	30–100	10–100
*EQ-5D utility*							
n	352	73	28	111	22	32	86
Mean (SD)	0.831 (0.206)	0.786 (0.275)	0.899 (0.231)	0.868 (0.132)	0.884 (0.100)	0.817 (0.176)	0.789 (0.222)
Median	0.889	0.889	0.985	0.889	0.890	0.890	0.812
Range	− 0.217–0.989	− 0.217–0.989	− 0.131–0.989	0.377–0.989	0.682–0.989	0.262–0.989	− 0.196–0.989

© 2009 EuroQol Research Foundation. EQ-5D™ is a trade mark of the EuroQol Research Foundation

ALS, amyotrophic lateral sclerosis; EoE, eosinophilic esophagitis; GvHD, graft versus host disease; HD, Huntington’s disease; MG, myasthenia gravis; PSP, progressive supranuclear palsy; SD, standard deviation; VAS, visual analogue scale

Most caregivers reported no problems for the EQ-5D-5L dimensions mobility, self-care and usual activities. However, only 48% reported no problem with anxiety/depression and 65% no problem with pain/discomfort. Twenty-five percent of caregivers reported slight pain/discomfort, 33% slight anxiety/depression, and 19% at least moderate anxiety and depression. The distribution of responses across EQ-5D-5L dimensions was similar for each of the diseases, although caregivers of care recipients with ALS, PSP and MG reported slightly greater problems than other diseases across all dimensions, and particularly for anxiety/depression.

Both EQ VAS scores and EQ-5D utilities showed large ranges, particularly when care recipients had ALS or PSP, indicating a great deal of variability in caregiver HRQoL. On the EQ VAS, some caregivers in all diseases scored themselves as 100, while others scored themselves close to 0. Some caregivers of care recipients with ALS, EoE and PSP had an EQ-5D utility below zero, while at least one caregiver in each disease group had a utility close to 1. Mean EQ VAS scores and EQ-5D utilities indicated worse caregiver HRQoL when care recipients had ALS or PSP, compared with other diseases.

### Caregiver employment, productivity and activity impairment

Data on caregivers’ employment and productivity are reported in Table 5.

**Table 5:** Caregiver employment and productivity

	Total (N = 365)	ALS (N = 75)	EoE (N = 29)	GvHD (N = 114)	HD (N = 26)	MG (N = 32)	PSP (N = 89)
*Caregiver currently employed, n (%)*							
n	330	72	0	114	25	31	88
Yes	148 (45%)	35 (49%)	–	59 (52%)	17 (68%)	10 (32%)	27 (31%)
No	182 (55%)	37 (51%)	–	55 (48%)	8 (32%)	21 (68%)	61 (69%)
*Time worked in last 7 days, hours*							
n	183	72	0	58	17	10	26
Mean (SD)	23.4 (16.2)	13.1 (18.0)	–	31.1 (9.3)	28.0 (12.7)	28.4 (12.6)	30.0 (11.4)
Median	25.0	0.0	–	33.5	30.0	33.5	34.5
Range	0–80	0–80	–	12–45	0–40	0–40	0–40
*Effect on caregiver’s employment*^*a,b*^*, n (%)*							
n	131	35	0	59	0	10	27
Reduced working hours	29 (22%)	10 (29%)	–	11 (19%)	–	2 (20%)	6 (22%)
Work from home	12 (9%)	12 (34%)	–	0 (0%)	–	0 (0%)	0 (0%)
Stopped working	2 (2%)	1 (3%)	–	0 (0%)	–	1 (10%)	0 (0%)
Changed job/type of work	4(3%)	2 (6%)	–	2 (3%)	–	0 (0%)	0 (0%)
Changed working hours	2 (2%)	2 (6%)	–	0 (0%)	–	0 (0%)	0 (0%)
No changes to employment	85 (65%)	11 (31%)	–	46 (78%)	–	7 (70%)	21 (78%)
*Reduction in caregiver’s income*^*b*^*, %*							
n	56	0	1	21	0	6	28
Mean (SD)	23.5 (22.3)	–	10.0 (0.0)	25.0 (23.1)	–	26.7 (10.3)	22.1 (24.2)
Median	22.5	–	10.0	25.0	–	30.0	20.0
Range	0–100	–	10–10	0–100	–	10–40	0–100
*Work time missed in last 7 days*^*b*^*, hours*							
n	175	73	0	51	15	10	26
Mean (SD)	1.2 (3.8)	1.2 (5.0)	–	(2.7)	0.5 (1.0)	(2.6)	2.0 (3.4)
Median	0	0.0	–	0.0	0.0	0.0	0.0
Range	0–40	0–40	–	0–10	0–3	0–8	0–14
*Absenteeism*^*c*^* in last 7 days*^*b*^*, %*							
n	128	31	0	51	13	9	24
Mean (SD)	5.2 (13.1)	9.4 (21.7)	–	3.5 (8.8)	1.5 (3.0)	3.6 (8.4)	6.1 (9.6)
Median	0.0	0.0	–	0.0	0.0	0.0	0.0
Range	0–100	0–100	–	0–33	0–8	0–25	0–37
*Presenteeism*^*d*^* in last 7 days*^*b*^*, %*							
n	141	33	0	59	15	9	25
Mean (SD)	28.0 (23.7)	33.6 (24.5)	–	25.1 (21.0)	34.7 (30.7)	26.7 (25.5)	24.0 (23.5)
Median	20.0	30.0	–	20.0	20.0	20.0	20.0
Range	0–100	0–90	–	0–80	0–100	0–70	0–80
*Overall work impairment*^*e*^* in last 7 days*^*b*^*, %*							
n	127	30	0	51	13	9	24
Mean (SD)	30.7 (26.1)	36.4 (27.5)	–	27.5 (24.2)	40.1 (30.2)	28.0 (27.4)	26.5 (25.3)
Median	20.0	27.5	–	20.0	30.0	20.0	20.0
Range	0–100	0–90	–	0–85	10–100	0–78	0–85
*Activity impairment*^*f*^* in last 7 days*^*b*^*, %*							
n	327	69	0	113	25	31	89
Mean (SD)	43.1 (27.2)	44.5 (29.3)	–	34.2 (21.5)	44.8 (28.2)	42.9 (26.5)	52.9 (28.8)
Median	40.0	40.0	–	30.0	40.0	40.0	50.0
Range	0–100	0–90	–	0–90	0–100	0–90	0–100

ALS, amyotrophic lateral sclerosis; EoE, eosinophilic esophagitis; GvHD, graft versus host disease; HD, Huntington’s disease; MG, myasthenia gravis; PSP, progressive supranuclear palsy; SD, standard deviation; WPAI, Work Productivity and Activity Impairment questionnaire

^a^Of those employed; ^b^Due to caregiving; ^c^Absenteeism = time missed from work; ^d^Presenteeism = impairment while at work; ^e^Overall work impairment = productivity loss due to absenteeism and presenteeism; ^f^Total activity impairment = limitation in daily activities outside of work

Overall, 45% of caregivers reported being currently employed, with a range of 31% in PSP to 68% in HD.

The overall mean hours worked by employed caregivers in the 7 days prior to completion of the WPAI was 23.4 (16.2), suggesting that few caregivers were in full-time employment. Caregivers for care recipients with ALS worked substantially lower mean and median hours (13.1 and 0.0, respectively) than caregivers in other diseases, but also had the greatest range (0–80).

The most common impact of caregiving on employment was reduced working hours, although among caregivers of individuals with ALS, working from home was reported slightly more frequently than reduced working hours. An overall mean reduction of 23.5% in caregivers’ income was reported; however, only 15% of caregivers provided this information.

Few hours of work were missed due to caregiving, and absenteeism was generally low, although the maximum reported absenteeism, for caregivers of individuals with ALS, was 100%. Impairment while working was a bigger issue than absenteeism, with caregivers reporting 28% mean presenteeism due to caregiving.

For caregivers completing the WPAI, mean overall work impairment was 31%, with mean activity impairment due to caregiving of 43%.

### Regression analyses

**Fig. 1:**
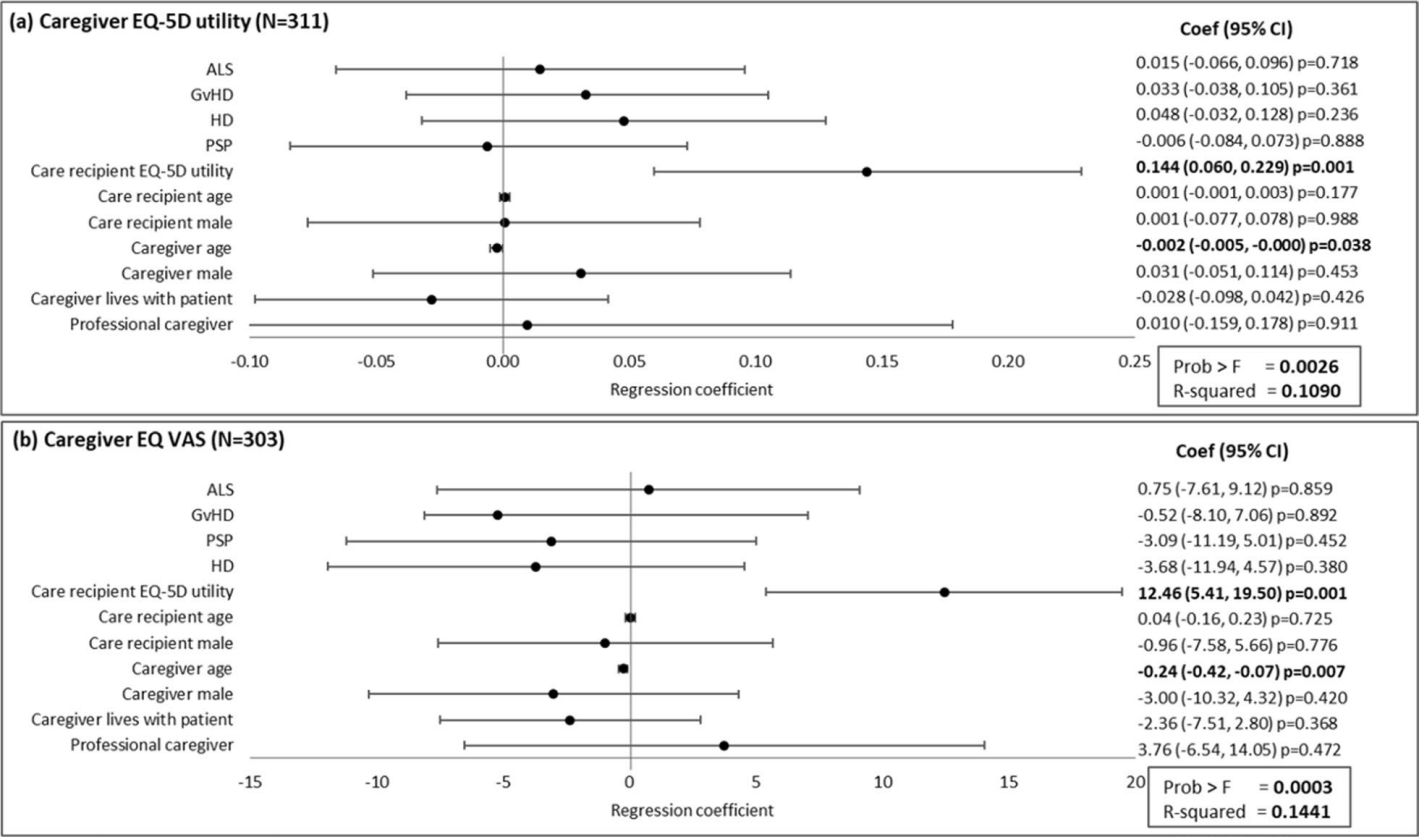
Association of caregiver and care recipient characteristics with caregiver health-related quality. Plots present the regression coefficient (95% CI). A regression coefficient significantly different from zero indicates a statistical association of the dependent and independent variable. ALS, amyotrophic lateral sclerosis; CI, confidence interval; GvHD, graft versus host disease; HD, Huntington’s disease; PSP, progressive supranuclear palsy; VAS, visual analogue scale

Regression analyses showed caregivers’ HRQoL, as defined by EQ-5D utility and EQ VAS score, was significantly associated with care recipient EQ-5D utility and caregivers’ age. Caregivers’ EQ-5D utility was directly correlated with care recipients’ EQ-5D utility (an increase of 10% in care recipient EQ-5D utility was associated with an increase of 1.4% in caregiver EQ-5D utility), while caregivers’ age was inversely correlated with caregivers’ EQ-5D utility (every additional year of caregivers’ age was associated with a reduction of 0.2% in caregivers’ EQ-5D utility) (Fig. 1a). An increase of 10% in care recipient EQ-5D utility was associated with an increase of 1.2% in caregiver EQ VAS score. Caregivers’ age was inversely correlated with caregivers’ EQ VAS score, with every additional year of caregivers’ age associated with a reduction of 0.2% in EQ VAS score (Fig. 1b).

**Fig. 2:**
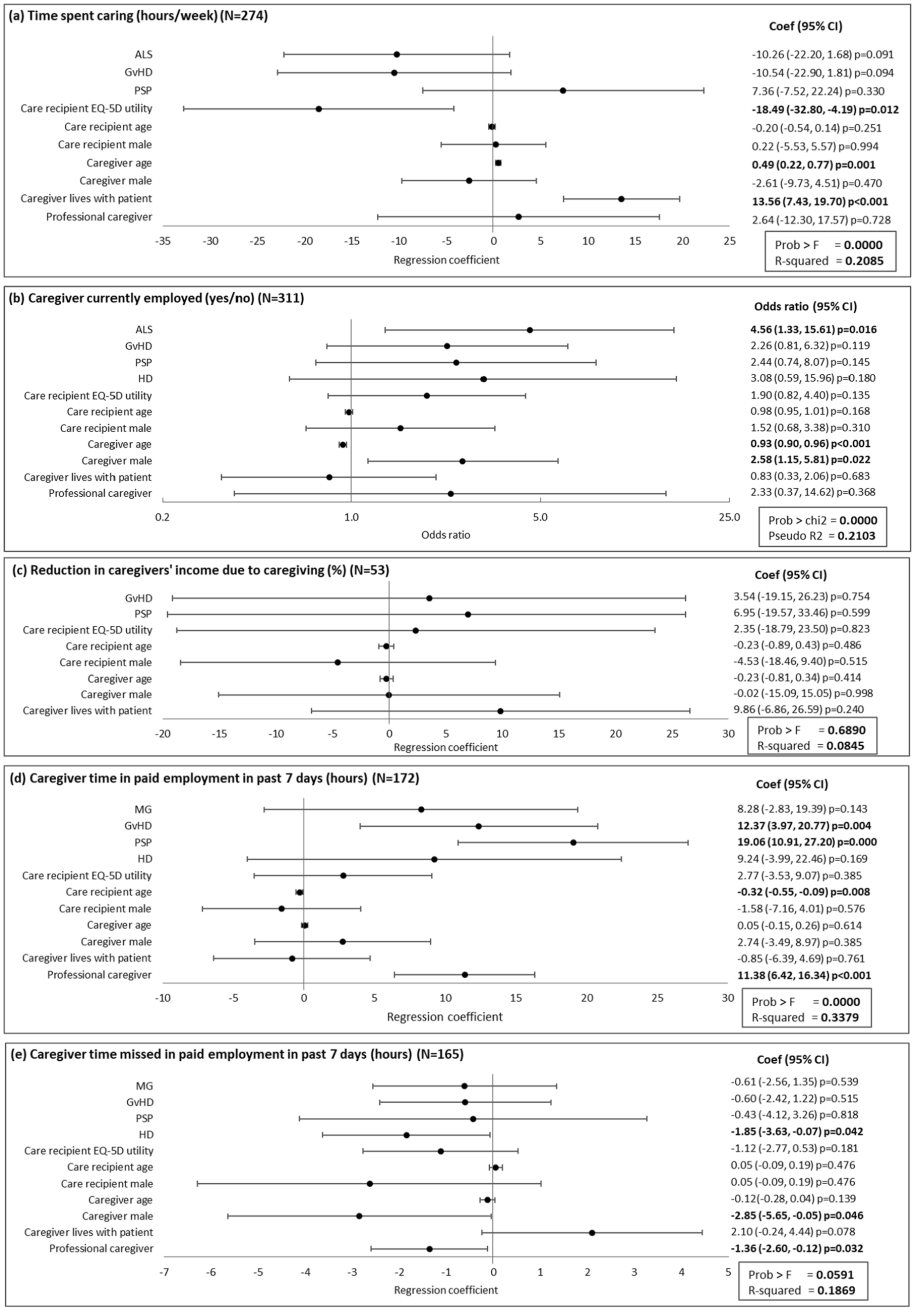
Association of caregiver and care recipient characteristics with caregiver time caring and employment characteristics. **a**, **c**, **d**, and **e** present the regression coefficient (95% CI) and **b** presents the odds ratio (95% CI). A regression coefficient significantly different from zero indicates a statistical association of the dependent and independent variable; an odds ratio significantly different from one indicates a statistical association of the dependent and independent variable. ALS, amyotrophic lateral sclerosis; CI, confidence interval; GvHD, graft versus host disease; HD, Huntington’s disease; MG, myasthenia gravis; PSP, progressive supranuclear palsy

Care recipients’ EQ-5D utility, caregivers’ age and living situation were all associated with the time caregivers spent caring. An increase of 10% (absolute) in care recipients’ EQ-5D utility equated to 1.8 h/week less caregiving time, every additional year of caregivers’ age equated to 0.5 additional hours caregiving time/week, and 13.6 more hours/week of caregiving was observed if the caregiver lived with the care recipient (Fig. 2a).

Caregivers’ age was inversely correlated with the odds that the caregiver was currently employed; if caregivers’ age decreased by 1 year, the odds the caregiver was employed increased by 7.9% (odds ratio of 0.93). The odds of being employed were 2.6 times higher for male versus female caregivers. ALS was associated with the odds of the caregiver being in employment, which was 4.6 times higher in caregivers of those with ALS than for other diseases (Fig. 2b). There were no significant associations with the reduction in caregivers’ income due to caregiving (Fig. 2c).

The age of the care recipient was significantly associated with caregiver time in paid employment in the past 7 days, with 0.3 fewer hours worked for every additional year of the care recipients’ age. Caregivers worked 11.4 more hours in the 7 days prior to data collection if the care recipient had a professional caregiver (as well as the non-professional caregiver providing the data). Regression analysis also showed that the care recipient having GvHD or PSP was significantly associated with the number of hours worked by the caregiver in the past 7 days (compared with other diseases, the caregiver worked an additional 12.4 h or 19.1 h, respectively) (Fig. 2d). Male caregivers missed 2.9 fewer hours in paid employment due to caregiving in the past 7 days than female caregivers. Caregivers also missed 1.4 fewer hours of work in the past 7 days if the care recipient had a professional caregiver. The care recipient having HD was significantly associated with the working time the caregiver missed due to caregiving in the past 7 days (compared with other diseases, the caregiver missed 1.9 fewer hours) (Fig. 2e).

**Fig. 3:**
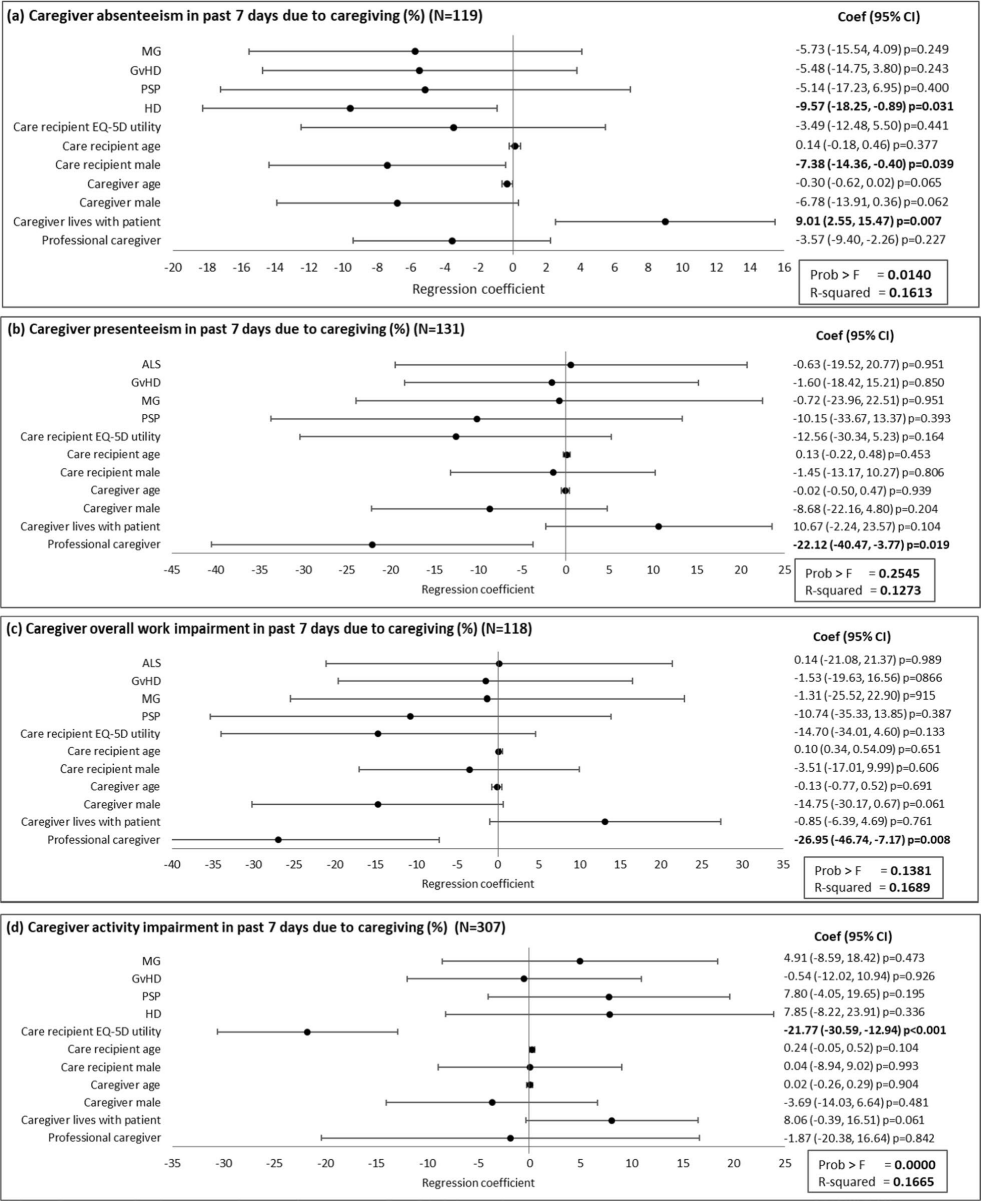
Association of caregiver and care recipient characteristics with caregiver productivity. Plots present the regression coefficient (95% CI). A regression coefficient significantly different from zero indicates a statistical association of the dependent and independent variable. ALS, amyotrophic lateral sclerosis; CI, confidence interval; GvHD, graft versus host disease; HD, Huntington’s disease; MG, myasthenia gravis; PSP, progressive supranuclear palsy

Caregivers’ absenteeism in the past 7 days was 7.4% lower if the care recipient was male rather than female and 9.0% higher if the caregiver lived with the care recipient. Caring for an adult with HD was associated with 9.6% lower absenteeism than caring for those with other diseases (Fig. 3a). Caregivers reported 22.1% lower presenteeism in the past 7 days and had 27.0% less overall work impairment in the past 7 days due to caregiving if the care recipient had a professional caregiver in addition to their non-professional caregiver (Fig. 3b, c). Regression analyses showed an increase of 10% (absolute) in care recipient EQ-5D utility equated to 2.2% less activity impairment for the caregiver in the past 7 days (Fig. 3d).

## Discussion

This analysis of real-world data from Europe and the USA investigated the HRQoL and productivity burden on non-professional caregivers of adults with rare diseases. The burden may differ from that of caring for people living with more common diseases, as fewer treatment options, the lack of disease-modifying drugs, poorly-established healthcare pathways and standards of care, and paucity of available information could all add to the caregivers’ burden. It may also differ from the burden experienced by parents caring for children, as the relationship between a parent and child and between two adults is likely to be different.

Our findings identified impairment of HRQoL in adults with rare diseases, and a substantial burden on their non-professional caregivers. Some caregivers reported impaired HRQoL, particularly in the area of anxiety/depression. The clearest issues related to caregivers’ productivity were presenteeism and impairment of out-of-work activities. We demonstrated the association of various factors with the HRQoL and productivity of non-professional caregivers of adults with different rare diseases.

Approximately one-quarter of care recipients had a professional caregiver in addition to their non-professional caregiver. Three-quarters of non-professional caregivers were the care recipient’s partner/spouse. These findings are consistent with outcomes of other studies across a range of rare diseases and in various countries [21, 22, 44].

Both the EQ VAS scores and the EQ-5D utilities suggest that care recipients with rare diseases have markedly poorer HRQoL than members of the general population. The overall mean EQ VAS score reported for patients was 50.9, substantially lower than population norms for participating countries, which range from 75.0 for Spain to 82.8 for the UK [39]. The only disease for which the care recipients’ EQ VAS score suggested no marked impairment of HRQoL was EoE, with a mean score of 82.3. The mean EQ VAS score for care recipients with ALS was 37.5, indicating major impairment of HRQoL. The overall mean EQ-5D utility for care recipients was 0.460, substantially lower than population norms for participating countries, which range from 0.825 (USA) to 0.915 (Spain) [39]. As with the EQ VAS score, the mean EQ-5D utility was highest for care recipients with EoE, although at 0.755, there was an indication of reduced HRQoL in this group. Care recipients with ALS and PSP had mean EQ-5D utilities of 0.276 and 0.277, respectively, signifying considerably poorer HRQoL than the general population.

The EQ-5D-5L has previously been shown to be appropriate for assessing caregivers’ health status in relation to the burden of caring in rare diseases [21, 45]. The overall mean EQ VAS score for caregivers was 81.6 and the mean EQ-5D utility was 0.831, indicating a quality of life similar to that of the general population in participating countries, with population norms for EQ VAS score ranging from 75.0 to 82.8 and EQ-5D utility from 0.825 to 0.915 [39]. However, similarity of caregiver and general population EQ VAS score and EQ-5D utility means does not indicate no impact of caregiving on HRQoL, since rare disease caregivers may differ from the general population in other respects.

The HRQoL of caregivers of adults with ALS or PSP was somewhat poorer than for other diseases. Although mean EQ VAS scores of 79.3 and 76.3 for ALS and PSP, respectively, do not indicate substantially poorer HRQoL than for the general population, mean EQ-5D utilities of 0.786 and 0.789 for ALS and PSP, respectively, are lower than those of the general population. This reflects the higher levels of problems reported across the dimensions, and particularly in anxiety/depression, for caregivers of adults with these diseases.

There is limited literature reporting HRQoL in caregivers of adults with rare diseases, especially across multiple conditions. A quantitative analysis of BURQOL-RD data for ten rare diseases from six European countries demonstrated impairment of caregivers’ HRQoL to be greater with higher caregiver burden (as assessed with the Zarit Burden Interview) [21]. However, the BURQOL-RD population comprised predominantly paediatric care recipients, and therefore HRQoL values are not directly comparable with those reported in our study. One study utilising data from a rare disease patient group in Hong Kong reported findings similar to ours, with a mean EQ VAS score of 76 and a mean EQ-5D utility of 0.80 [20]; population norms for China are 80.4 for EQ VAS score and 0.951 for EQ-5D utility [39]. Our findings that caregivers’ self-reported responses on the five dimensions of the EQ-5D-5L indicated some issues with anxiety or depression confirmed findings from other studies [20–22]. Additionally, evidence from published studies indicates that caregivers’ HRQoL varies by disease [29, 46–48] and country [49, 50].

Over half of caregivers were not currently employed. Many caregivers reported reduced working hours and income from their caregiving responsibilities. For employed caregivers, work time missed and percentage absenteeism due to caregiving were relatively low, but their presenteeism was marked. Activity impairment was considerable for caregivers regardless of employment status or care recipients’ disease. A literature search did not identify any studies of caregiver productivity across rare diseases or in any of the diseases included in this study. However, low levels of absenteeism, substantial presenteeism, and activity impairments have been reported in other studies of caregivers of adults with short bowel syndrome or lupus erythematosus [51, 52] and high indirect costs resulting from lost productivity have been reported based on data from the BURQOL-RD project [24].

We used regression analysis to model the relationship between care recipient and caregiver characteristics, and caregiver HRQoL and productivity. The independent variables that were significantly associated with the highest number of dependent variables were care recipients’ EQ-5D utility, caregivers’ age, and having a professional caregiver. Care recipients’ EQ VAS score was not included as an independent variable in the analyses as EQ VAS score and EQ-5D utility are both summary measures of HRQoL. More time caring and poorer caregiver HRQoL (lower EQ VAS score and EQ-5D utility) were observed with lower care recipient EQ-5D utility and greater caregiver age. Greater caregiver activity impairment was associated with poorer care recipients’ EQ-5D utility, and a reduced probability of the caregiver being employed was associated with greater caregiver age.

Regression analysis also showed that the care recipient having ALS was associated with a greater likelihood of the caregiver being employed, and the care recipient having GvHD or PSP was associated with the caregiver working longer hours. The caregiver living with the care recipient was associated with a higher number of hours caregiving, while the care recipient having a professional caregiver was associated with caregivers working longer hours, missing fewer hours of work, and lower presenteeism and overall work impairment.

Many of these associations might be expected intuitively. It makes sense that older caregivers had poorer HRQoL, were less likely to be in paid employment, and spent more time caring (they are less likely to be employed). It is also to be expected that having a professional caregiver will allow the main caregiver to spend more time working and have less work impairment. Finding that poorer care recipient HRQoL was associated with more time caring, greater caregiver activity impairment, and poorer caregiver HRQoL also appears logical. The associations of some specific diseases with particular outcomes are less obvious and may even appear counterintuitive. Caregivers for people with ALS were more likely to be working than those caring for people with other diseases, although care recipients with ALS tended to be older, have more severe disease and worse health status than those with other diseases. Compared to other diseases, caregivers in GvHD and PSP worked more hours, and caregivers in HD missed fewer work hours and had lower absenteeism. One possible explanation is that these findings relate to the age of caregivers and the relationship of care recipients and caregivers. Caregivers in EoE (the disease used as the reference disease in regression models) had the lowest mean age of any disease, and 25% of EoE caregivers were parents of the care recipients, compared with 2% overall. More than 70% of caregivers in ALS, GvHD, PSP and HD were the partner or spouse of the care recipient, compared with only 57% in EoE. In PSP, 24% of caregivers were the child of the care recipient, versus 4% in EoE. Therefore, it is possible that caregivers in ALS, GvHD, PSP and HD had more opportunity to establish a career prior to providing care compared with caregivers in EoE, leading to a higher likelihood of being employed, the possibility of working longer hours, and fewer working hours missed in these diseases versus EoE.

A few published studies have investigated the association between the HRQoL of adults with rare diseases and their caregivers’ HRQoL and productivity. The Hong Kong rare disease study reported correlations between caregivers’ and care recipients’ EQ-5D utility (*p* = 0.001) [20] and a study in cystic fibrosis in France identified strong correlations between care recipient EQ-5D utility and caregiver utility (*p* = 0.003) and VAS score (*p* < 0.0001 [29], aligning with our finding of lower caregiver EQ VAS score and utility with lower care recipient EQ-5D utility.

Our study had several potential limitations. The requirement for care recipients to consult their physician means the study sample was potentially not representative of the wider rare disease population; care recipients who consulted their physician more frequently than average might be over-represented. Despite the high numbers of study participants, missing data resulted in small sample sizes for some variables. As with all studies of this type, the methodology relied on accurate reporting—missing data were to be expected but may have influenced results. Variables with most missing values were those not commonly recorded in patient records or discussed during consultations, such as the time spent caring or caregiver productivity. Missing data are not unusual in observational studies, and, due to the generally low proportion of missing independent variables in regression models, no statistical approach was used to replace missing values. However, the possibility of a degree of bias due to missing data cannot be excluded. As this was a cross-sectional survey, the analyses explored the association between variables but not causality. The fact that the caregiver completed the EQ-5D-5L both for themselves and as a proxy for the patient might result in greater caregiver/patient HRQoL correlation than would be the case if the patient reported their own HRQoL, although the use of proxy-reported HRQoL is widely accepted when considering populations where physical or cognitive impairment limits self-reporting [53]. In assessing the impact on caregiver productivity, only paid work was considered; productivity loss did not include opportunity cost for caregivers who were not employed. Finally, the fact that we combined data from six rare diseases, with differing characteristics, might be considered a limitation. However, we controlled for disease in the regression models to allow for the heterogeneous study population. As there are few studies evaluating the impact of rare diseases across a range of conditions, we believe this is a potential advantage of the study, with outcomes generalisable beyond one disease area. Despite any potential limitations, this study provides valuable insight into factors influencing the HRQoL and productivity of caregivers of adults with rare diseases.

Study outcomes indicate that rare diseases can be associated with substantial detriments to caregivers' HRQoL and productivity, with implications for the provision of health and related services. It is important to consider the impact of interventions on carers as well as patients. There are also implications for economic modelling, as evaluations that take a traditional healthcare system perspective and limit assessment to patients’ health outcomes are potentially overlooking a considerable HRQoL and economic impact on caregivers. In particular, our findings indicate that improvements in patients' HRQoL may be associated with improvements in caregivers' HRQoL and productivity.

## Conclusions

In conclusion, the HRQoL and productivity burden on those providing non-professional caregiving in rare diseases is considerable. Many published studies of caregiving in rare diseases focus on caring for children; this study shows that there is a substantial burden in adults caring for other adults across a range of rare diseases and in a number of countries. The burden on caregivers is associated with a number of factors, some of which, such as caregiver age, cannot be managed. However, it appears that any intervention that improves care recipient HRQoL could potentially improve caregiver HRQoL and productivity. The prevalence of rare diseases, the observed burden for non-professional caregivers, and the paucity of published studies examining these hypotheses suggest further research is warranted. Investigating similar outcomes with paediatric patients with rare diseases, or with a focus on inherited diseases, could generate interesting, relevant and actionable findings.

## Supplementary Information

Additional file 1.

## Data Availability

All data, i.e. methodology, materials, data and data analysis, that support the findings of this survey are the intellectual property of Adelphi Real World. All requests for access should be addressed directly to Jonathan de Courcy at jonathan.decourcy@adelphigroup.com. Jonathan de Courcy is an employee of Adelphi Real World.

## Abbreviations

ALS: Amyotrophic lateral sclerosis
CI: Confidence interval
DSP: Disease Specific Programme
EoE: Eosinophilic esophagitis
EQ VAS: EuroQol visual analogue scale
GvHD: Graft versus host disease
HD: Huntington’s disease
HRQoL: Health-related quality of life
MG: Myasthenia gravis
PSP: Progressive supranuclear palsy
SD: Standard deviation
USA: United States of America
VAS: Visual analogue scale
VIF: Variance inflation factors
WPAI: Work productivity and activity impairment

## References

[1] Richter T, Nestler-Parr S, Babela R, Khan ZM, Tesoro T, Molsen E, Hughes DA, International Society for Pharmacoeconomics and Outcomes Research Rare Disease Special Interest Group. Rare disease terminology and definitions—a systematic global review: report of the ISPOR rare disease special interest group. Value Health. 2015;18(6):906–14. 10.1016/j.jval.2015.05.008. 26409619 10.1016/j.jval.2015.05.008

[2] Cui Y, Han J. A proposed definition of rare diseases for China: from the perspective of return on investment in new orphan drugs. Orphanet J Rare Dis. 2015;10:28. 10.1186/s13023-015-0241-x. 25757391 10.1186/s13023-015-0241-x PMC4353681

[3] Abozaid GM, Kerr K, McKnight A, Al-Omar HA. Criteria to define rare diseases and orphan drugs: a systematic review protocol. BMJ Open. 2022;12(7): e062126. 10.1136/bmjopen-2022-062126. 35906057 10.1136/bmjopen-2022-062126 PMC9345065

[4] Nguengang Wakap S, Lambert DM, Olry A, Rodwell C, Gueydan C, Lanneau V, Murphy D, Le Cam Y, Rath A. Estimating cumulative point prevalence of rare diseases: analysis of the Orphanet database. Eur J Hum Genet. 2020;28(2):165–73. 10.1038/s41431-019-0508-0. 31527858 10.1038/s41431-019-0508-0 PMC6974615

[5] Pavan S, Rommel K, Mateo Marquina ME, Höhn S, Lanneau V, Rath A. Clinical practice guidelines for rare diseases: the orphanet database. PLoS ONE. 2017;12(1): e0170365. 10.1371/journal.pone.0170365. 28099516 10.1371/journal.pone.0170365 PMC5242437

[6] Smit C. Ageing in rare, chronic diseases. Orphanet J Rare Dis. 2010;5(Suppl 1):O23. 10.1186/1750-1172-5-S1-O23.

[7] Rusz CM, Ősz BE, Jîtcă G, Miklos A, Bătrînu MG, Imre S. Off-label medication: from a simple concept to complex practical aspects. Int J Environ Res Public Health. 2021;18(19):10447. 10.3390/ijerph181910447. 34639747 10.3390/ijerph181910447 PMC8508135

[8] European Union (EU). Regulation (EC) N°141/2000 of the European Parliament and of the Council of 16 December 1999 on orphan medicinal products. 2000. http://eurlex.europa.eu/LexUriServ/LexUriServ.do?uri=OJ:L:2000:018:0001:0005:EN:PDF. Accessed 28 Sep 2022.

[9] Shiragami M, Nakai K. Development of orphan drugs in Japan: effects of a support system for development of orphan drugs in japan. Drug Inf J. 2000;34(3):829–37. 10.1177/009286150003400319.

[10] Song P, Gao J, Inagaki Y, Kokudo N, Tang W. Rare diseases, orphan drugs, and their regulation in Asia: current status and future perspectives. Intractable Rare Dis Res. 2012;1(1):3–9. 10.5582/irdr.2012.v1.1.3. 25343064 10.5582/irdr.2012.v1.1.3 PMC4204590

[11] Department of Health (DoH Australia). Orphan drug designation eligibility criteria. 2017; updated 23 April 2021. https://www.tga.gov.au/publication/orphan-drug-designationeligibility-criteria. Accessed 28 Sep 2022.

[12] Lord BRS, Vaughan G, Mairi G. The UK rare diseases framework, 2021. https://www.gov.uk/government/publications/ukrare-diseases-framework/the-uk-rare-diseases-framework. Accessed 28 Sep 2022.

[13] National Institute of Health (NIH). Public Law 107–280 107th Congress. Nov 6, 2002. https://www.congress.gov/107/plaws/publ280/PLAW-107publ280.pdf. Accessed 28 Sep 2022.

[14] Food and Drug Administration (FDA). Rare Diseases: Common Issues in Drug Development Guidance for Industry. January 2019. https://www.fda.gov/media/119757/download. Accessed 4 Oct 2022.

[15] US Department of Commerce (US DoC). U.S. Population Estimated at 332,403,650 on Jan. 1, 2022. https://www.commerce.gov/data-and-reports/population-statistics. Accessed 29 Sep 2022.

[16] Haendel M, Vasilevsky N, Unni D, Bologa C, Harris N, Rehm H, Hamosh A, Baynam G, Groza T, McMurry J, Dawkins H, Rath A, Thaxon C, Bocci G, Joachimiak MP, Köhler S, Robinson PN, Mungall C, Oprea TI. How many rare diseases are there? Nat Rev Drug Discov. 2020;19(2):77–8. 10.1038/d41573-019-00180-y. 32020066 10.1038/d41573-019-00180-y PMC7771654

[17] European Commission (EC). CEU Research on Rare Diseases. https://research-and-innovation.ec.europa.eu/research-area/health/rare-diseases_en. Accessed 29 Sep 2022.

[18] Genetic and Rare Diseases Information Center (GARD). You Are Not Alone. https://rarediseases.info.nih.gov/. Accessed 29 Sep 2022.

[19] Lenderking WR, Anatchkova M, Pokrzywinski R, Skalicky A, Martin ML, Gelhorn H. Measuring health-related quality of life in patients with rare disease. J Patient Rep Outcomes. 2021;5(1):61. 10.1186/s41687-021-00336-8. 34283357 10.1186/s41687-021-00336-8 PMC8292508

[20] Ng YNC, Ng NYT, Fung JLF, Lui ACY, Cheung NYC, Wong WHS, Lee SL, Knapp M, Chung CCY, Chung BHY. Evaluating the health-related quality of life of the rare disease population in Hong Kong using EQ-5D 3-level. Value Health. 2022;25(9):1624–33. 10.1016/j.jval.2022.04.1725. 35568675 10.1016/j.jval.2022.04.1725

[21] Valcárcel-Nazco C, Ramallo-Fariña Y, Linertová R, Ramos-Goñi JM, García-Pérez L, Serrano-Aguilar P. Health-related quality of life and perceived burden of informal caregivers of patients with rare diseases in selected European countries. Int J Environ Res Public Health. 2022;19(13):8208. 10.3390/ijerph19138208. 35805867 10.3390/ijerph19138208 PMC9266302

[22] Xu J, Bao H, Qi X, Wang J, Yin H, Shang C, Tan RL, Wu Q, Huang W. Family caregivers of rare disease: a survey on health-related quality of life in family caregivers for Gaucher disease patients in China. Mol Genet Genomic Med. 2021;9(9): e1760. 10.1002/mgg3.1760. 34387413 10.1002/mgg3.1760 PMC8457695

[23] Yang G, Cintina I, Pariser A, Oehrlein E, Sullivan J, Kennedy A. The national economic burden of rare disease in the United States in 2019. Orphanet J Rare Dis. 2022;17(1):163. 10.1186/s13023-022-02299-5. 35414039 10.1186/s13023-022-02299-5 PMC9004040

[24] López-Bastida J, Oliva-Moreno J, Linertová R, Serrano-Aguilar P. Social/economic costs and health-related quality of life in patients with rare diseases in Europe. Eur J Health Econ. 2016;17(Suppl 1):1–5. 10.1007/s10198-016-0780-7. 27023708 10.1007/s10198-016-0780-7

[25] Antoniadi AM, Galvin M, Heverin M, Hardiman O, Mooney C. Prediction of caregiver quality of life in amyotrophic lateral sclerosis using explainable machine learning. Sci Rep. 2021;11(1):12237. 10.1038/s41598-021-91632-2. 34112871 10.1038/s41598-021-91632-2 PMC8192926

[26] McMillan HJ, Gerber B, Cowling T, Khuu W, Mayer M, Wu JW, Maturi B, Klein-Panneton K, Cabalteja C, Lochmüller H. Burden of spinal muscular atrophy (SMA) on patients and caregivers in Canada. J Neuromuscul Dis. 2021;8(4):553–68. 10.3233/JND-200610. 33749617 10.3233/JND-200610 PMC8385498

[27] Kodra Y, Cavazza M, de Santis M, Guala A, Liverani ME, Armeni P, Masini M, Taruscio D. Social economic costs, health-related quality of life and disability in patients with Cri Du chat syndrome. Int J Environ Res Public Health. 2020;17(16):5951. 10.3390/ijerph17165951. 32824402 10.3390/ijerph17165951 PMC7459640

[28] Péntek M, Herczegfalvi Á, Molnár MJ, Szőnyi LP, Kosztolányi G, Pfliegler G, Melegh B, Boncz I, Brodszky V, Baji P, Szegedi M, Pogány G, Gulácsi L. DUCHENNE-FÉLE IZOMDISZTRÓFIÁVAL ÉLO BETEGEK ÉS GONDOZÓIK BETEGSÉGTERHEI [DISEASE BURDEN OP DUCHENNE MUSCULAR DYSTROPHY PATIENTS AND THEIR CAREGIVERS]. Ideggyogy Sz. 2016;69(5–6):183–93. 10.18071/isz.69.0183. 10.18071/isz.69.0183 27468608

[29] Chevreul K, BergBrigham K, Michel M, Rault G, BURQOL-RD Research Network. Costs and health-related quality of life of patients with cystic fibrosis and their carers in France. J Cyst Fibros. 2015;14(3):384–91. 10.1016/j.jcf.2014.11.006. 25620688 10.1016/j.jcf.2014.11.006

[30] da Silva LB, Ivo ML, de Souza AS, Pontes ER, Pinto AM, de Araujo OM. The burden and quality of life of caregivers of sickle cell anemia patients taking hydroxyurea versus those not taking hydroxyurea. Rev Bras Hematol Hemoter. 2012;34(4):270–4. 10.5581/1516-8484.20120070. 23049439 10.5581/1516-8484.20120070 PMC3460397

[31] Somanadhan S, Larkin PJ. Parents’ experiences of living with, and caring for children, adolescents and young adults with Mucopolysaccharidosis (MPS). Orphanet J Rare Dis. 2016;11(1):138. 10.1186/s13023-016-0521-0. 27724940 10.1186/s13023-016-0521-0 PMC5057247

[32] Lagae L, Irwin J, Gibson E, Battersby A. Caregiver impact and health service use in high and low severity Dravet syndrome: a multinational cohort study. Seizure. 2019;65:72–9. 10.1016/j.seizure.2018.12.018. 30616222 10.1016/j.seizure.2018.12.018

[33] Stewart M, Shaffer S, Murphy B, Loftus J, Alvir J, Cicchetti M, Lenderking WR. Characterizing the high disease burden of transthyretin amyloidosis for patients and caregivers. Neurol Ther. 2018;7(2):349–64. 10.1007/s40120-018-0106-z. 30073497 10.1007/s40120-018-0106-z PMC6283802

[34] Linertová R, Serrano-Aguilar P, Posada-de-la-Paz M, Hens-Pérez M, Kanavos P, Taruscio D, Schieppati A, Stefanov R, Péntek M, Delgado C, von der Schulenburg JM, Persson U, Chevreul K, Fattore G, Worbes-Cerezo M, Sefton M, López-Bastida J; BURQOL-RD Research Group. Delphi approach to select rare diseases for a European representative survey. The BURQOL-RD study. Health Policy. 2012;108(1):19–26. 10.1016/j.healthpol.2012.08.001. 10.1016/j.healthpol.2012.08.001 22947412

[35] Anderson P, Benford M, Harris N, Karavali M, Piercy J. Real-world physician and patient behaviour across countries: disease-Specific Programmes—a means to understand. Curr Med Res Opin. 2008;24(11):3063–72. 10.1185/03007990802457040. 18826746 10.1185/03007990802457040

[36] EuroQoL. EQ-5D-5L. https://euroqol.org/eq-5d-instruments/eq-5d-5l-about/. Accessed 31 Oct 2022.

[37] Hernández-Alava M, Pudney S. EQ5Dmap: a command for mapping between EQ-5D-3L and EQ-5D-5L. Stand Genomic Sci. 2018;18(2):395–415. 10.1177/1536867X1801800207.

[38] Hernández Alava M, Pudney S, Wailoo A. Estimating the relationship between EQ-5D-5L and EQ-5D-3L: results from a UK population study. Pharmacoeconomics. 2023;41(2):199–207. 10.1007/s40273-022-01218-7. 36449173 10.1007/s40273-022-01218-7 PMC9883358

[39] Janssen B, Szende A. Population norms for the EQ-5D. In: Szende A, Janssen B, Cabases J, editors. Self-reported population health: an international perspective based on EQ-5D. Dordrecht: Springer; 2013. p. 2014. 29787044

[40] Dawoud D, Lamb A, Moore A, Bregman C, Rupniewska E, Paling T, Wolfram V, Lovett RES, Dent R. Capturing what matters: updating NICE methods guidance on measuring and valuing health. Qual Life Res. 2022;31(7):2167–73. 10.1007/s11136-022-03101-6. 35247152 10.1007/s11136-022-03101-6 PMC9188493

[41] Reilly MC, Zbrozek AS, Dukes EM. The validity and reproducibility of a work productivity and activity impairment instrument. Pharmacoeconomics. 1993;4(5):353–65. 10.2165/00019053-199304050-00006. 10146874 10.2165/00019053-199304050-00006

[42] Hocking RR. Developments in linear regression methodology: 1959–1982. Technometrics. 1983;25(3):219–30. 10.1080/00401706.1983.10487871.

[43] StataCorp. Stata statistical software: release 17. College Station: StataCorp LLC; 2021.

[44] McMullan J, Crowe AL, Downes K, McAneney H, McKnight AJ. Carer reported experiences: supporting someone with a rare disease. Health Soc Care Community. 2022;30(3):1097–108. 10.1111/hsc.13336. 33955634 10.1111/hsc.13336

[45] Crossnohere NL, Fischer R, Lloyd A, Prosser LA, Bridges JFP. Assessing the appropriateness of the EQ-5D for Duchenne muscular dystrophy: a patient-centered study. Med Decis Mak. 2021;41(2):209–21. 10.1177/0272989X20978390. 10.1177/0272989X20978390 33463405

[46] Péntek M, Baji P, Pogány G, Brodszky V, Boncz I, Gulácsi L. Health related quality of life of patients and their caregivers in rare diseases results of the BURQOL-RD project In Hungary. Value Health. 2014;17(7):A538. 10.1016/j.jval.2014.08.1724. 27201723 10.1016/j.jval.2014.08.1724

[47] Angelis A, Kanavos P, López-Bastida J, Linertová R, Serrano-Aguilar P, BURQOL-RD Research Network. Socioeconomic costs and health-related quality of life in juvenile idiopathic arthritis: a cost-of-illness study in the United Kingdom. BMC Musculoskelet Disord. 2016;17:321. 10.1186/s12891-016-1129-1. 27484740 10.1186/s12891-016-1129-1 PMC4971720

[48] Angelis A, Kanavos P, López-Bastida J, Linertová R, Nicod E, Serrano-Aguilar P, BURQOL-RD Research Network. Social and economic costs and health-related quality of life in non-institutionalised patients with cystic fibrosis in the United Kingdom. BMC Health Serv Res. 2015;15:428. 10.1186/s12913-015-1061-3. 26416027 10.1186/s12913-015-1061-3 PMC4587726

[49] Péntek M, Gulácsi L, Brodszky V, Baji P, Boncz I, Pogány G, López-Bastida J, Linertová R, Oliva-Moreno J, Serrano-Aguilar P, Posada-de-la-Paz M, Taruscio D, Iskrov G, Schieppati A, von der Schulenburg JM, Kanavos P, Chevreul K, Persson U, Fattore G, BURQOL-RD Research Network. Social/economic costs and health-related quality of life of mucopolysaccharidosis patients and their caregivers in Europe. Eur J Health Econ. 2016;17(Suppl 1):89–98. 10.1007/s10198-016-0787-0. 27062257 10.1007/s10198-016-0787-0

[50] Angelis A, Kanavos P, López-Bastida J, Linertová R, Oliva-Moreno J, Serrano-Aguilar P, Posada-de-la-Paz M, Taruscio D, Schieppati A, Iskrov G, Brodszky V, von der Schulenburg JM, Chevreul K, Persson U, Fattore G, BURQOL-RD Research Network. Social/economic costs and health-related quality of life in patients with epidermolysis bullosa in Europe. Eur J Health Econ. 2016;17(Suppl 1):31–42. 10.1007/s10198-016-0783-4. 27107597 10.1007/s10198-016-0783-4 PMC4869727

[51] Jeppesen PB, Chen K, Murphy R, Shahraz S, Goodwin B. Impact on caregivers of adult patients receiving parenteral support for short-bowel syndrome with intestinal failure: a multinational, cross-sectional survey. JPEN J Parenter Enteral Nutr. 2022;46(4):905–14. 10.1002/jpen.2248. 34368993 10.1002/jpen.2248 PMC9293039

[52] Al Sawah S, Daly RP, Foster SA, Naegeli AN, Benjamin K, Doll H, Bond G, Moshkovich O, Alarcón GS. The caregiver burden in lupus: findings from UNVEIL, a national online lupus survey in the United States. Lupus. 2017;26(1):54–61. 10.1177/0961203316651743. 27235701 10.1177/0961203316651743

[53] Rajmil L, Perestelo-Pérez L, Herdman M. Quality of life and rare diseases. Adv Exp Med Biol. 2010;686:251–72. 10.1007/978-90-481-9485-8_15. 20824450 10.1007/978-90-481-9485-8_15

