# Supplementary material for: Health-related quality of life and productivity burden for non-professional caregivers of adults with rare diseases: a real-world study
Source: Orphanet J Rare Dis. 2025 Jun 6;20:282. doi: 10.1186/s13023-025-03796-z (PMC12142911; doi:10.1186/s13023-025-03796-z)
Supplement: Supplementary file 1 — Additional file 1. [file 13023_2025_3796_MOESM1_ESM.docx]

**Table S1 Disease overview**

|  | **Epidemiology** | **Treatment options** | **Age at onset** | **Life expectancy** | **Symptoms/**  **Disease progression** |
| --- | --- | --- | --- | --- | --- |
| Huntington’s disease | Global incidence: 0.48/100,000/year^1^  Global prevalence: 4.88/100,000^1^ | Treatment focused on controlling symptoms^2^  Tetrabenazine licensed for treatment of chorea in HD^2^ | Generally mid-life;  Young-onset <21 years;  Late-onset can be in 80s^2^ | 15-20 years after diagnosis^3^ | Neuropsychiatric symptoms, progressive movement disorder, dementia^2^ |
| Progressive supranuclear palsy | Global prevalence: 5‑6/100,000^4^ | Symptomatic treatment only^5^ | Mean age 63^4^ | Median survival 7 years^4^ | Variable clinical course;  Declines in motor/ oculomotor functions & activities of daily living^6^ |
| Graft versus host disease | USA: 42% develop chronic GVHD within 3 years^7^ | Corticosteroids most common treatment^7^ | N/A | For survivors 2 years post-transplant – 10 year survival of 91.9%^8^ | Oral manifestations often are first symptom^9^ multi‐organ pathology; commonly skin, mouth, eyes, joints, genitals, gastro-intestinal tract, liver, and lungs^10^ |
| Myasthenia gravis | Global incidence: 0.53/100,000/year^11^  Global prevalence: 1.5-36.7/100,000^11^ | Pyridostigmine, corticosteroids, immunosuppressants^11^ | Females: 20 and 39 years ; Males 50 and 70 years Bimodal onset in females with early and late-onset peaks, one late-onset peak for males^11^ | Normal life expectancy^13^ | Manifests as fatigue and fluctuating weakness of striated muscles, which progresses.^12^ |
| Eosinophilic esophagitis | Global incidence: 6.6/100,000/year (children); 7.7/100,000/year (adults).  Global prevalence: 34.4/100,000. ^14^ | Proton pump inhibitors; topical steroids^15^ | Age at diagnosis 5.9–12.0 years in children, ~30 years in adults^16^ | Normal life expectancy^17^ | Esophageal dysfunction and eosinophilic histologic inflammation.  Untreated disease can lead to esophageal remodeling and strictures^18^ |
| Amyotrophic lateral sclerosis | Europe incidence:  1.75–3/100,000/year  Europe prevalence: 10–12/100,000^19^ | Symptomatic treatment only^19^ | Mean age at symptom onset: 58–63 years for sporadic ALS; 40–60 years for familial ALS^19^ | 3-5 years post diagnosis^20^ | Progressive loss of motor neurons in the brain and spinal cord^21^ |

**References**

1. Medina A, Mahjoub Y, Shaver L, Pringsheim T. Prevalence and Incidence of Huntington's Disease: An Updated Systematic Review and Meta-Analysis. Mov Disord. 2022 Dec;37(12):2327-2335. doi: 10.1002/mds.29228. Epub 2022 Sep 26. PMID: 36161673; PMCID: PMC10086981.
2. Stoker TB, Mason SL, Greenland JC, Holden ST, Santini H, Barker RA. Huntington's disease: diagnosis and management. Pract Neurol. 2022 Feb;22(1):32-41. doi: 10.1136/practneurol-2021-003074. Epub 2021 Aug 19. PMID: 34413240.
3. Boersema-Wijma DJ, van Duijn E, Heemskerk AW, van der Steen JT, Achterberg WP. Palliative care in advanced Huntington's disease: a scoping review. BMC Palliat Care. 2023 May 3;22(1):54. doi: 10.1186/s12904-023-01171-y. Erratum in: BMC Palliat Care. 2023 May 31;22(1):63. PMID: 37138329; PMCID: PMC10155365.
4. Golbe LI. Progressive supranuclear palsy. Semin Neurol. 2014 Apr;34(2):151-9. doi: 10.1055/s-0034-1381736. Epub 2014 Jun 25. PMID: 24963674.
5. Lamb R, Rohrer JD, Lees AJ, Morris HR. Progressive Supranuclear Palsy and Corticobasal Degeneration: Pathophysiology and Treatment Options. Curr Treat Options Neurol. 2016 Sep;18(9):42. doi: 10.1007/s11940-016-0422-5. PMID: 27526039; PMCID: PMC4985534.
6. Litvan I, Kong M. Rate of decline in progressive supranuclear palsy. Mov Disord. 2014 Apr;29(4):463-8. doi: 10.1002/mds.25843. Epub 2014 Feb 24. PMID: 24615741.
7. Bachier CR, Aggarwal SK, Hennegan K, Milgroom A, Francis K, Dehipawala S, Rotta M. Epidemiology and Treatment of Chronic Graft-versus-Host Disease Post-Allogeneic Hematopoietic Cell Transplantation: A US Claims Analysis. Transplant Cell Ther. 2021 Jun;27(6):504.e1-504.e6. doi: 10.1016/j.jtct.2020.12.027. Epub 2020 Dec 31. PMID: 34158154.
8. Wu L, Wu Y, Shi J, Lai X, Zhao Y, Liu L, Yu J, Yang L, Zhu P, Zheng W, Hu Y, Wu W, Zhu Y, Cai Z, Huang H, Luo Y. Survival and late mortality among patients who survived disease-free for 2 years after stem cell transplantation. Br J Haematol. 2023 Aug;202(3):608-622. doi: 10.1111/bjh.18905. Epub 2023 Jun 12. PMID: 37306071.
9. Tollemar V, Garming Legert K, Sugars RV. Perspectives on oral chronic graft-versus-host disease from immunobiology to morbid diagnoses. Front Immunol. 2023 Jun 28;14:1151493. doi: 10.3389/fimmu.2023.1151493. PMID: 37449200; PMCID: PMC10338056.
10. Yu J, Hamilton BK, Turnbull J, Stewart SK, Vernaya A, Bhatt V, Meyers O, Galvin J. Patient-reported symptom burden and impact on daily activities in chronic graft-versus-host disease. Cancer Med. 2023 Feb;12(3):3623-3633. doi: 10.1002/cam4.5209. Epub 2022 Nov 16. PMID: 36394207; PMCID: PMC9939096.
11. Bubuioc AM, Kudebayeva A, Turuspekova S, Lisnic V, Leone MA. The epidemiology of myasthenia gravis. J Med Life. 2021 Jan-Mar;14(1):7-16. doi: 10.25122/jml-2020-0145. PMID: 33767779; PMCID: PMC7982252.
12. Estephan EP, Baima JPS, Zambon AA. Myasthenia gravis in clinical practice. Arq Neuropsiquiatr. 2022 May;80(5 Suppl 1):257-265. doi: 10.1590/0004-282X-ANP-2022-S105. PMID: 35976295; PMCID: PMC9491427.
13. Juel VC, Massey JM. Myasthenia gravis. Orphanet J Rare Dis. 2007 Nov 6;2:44. doi: 10.1186/1750-1172-2-44. PMID: 17986328; PMCID: PMC2211463.
14. Navarro P, Arias Á, Arias-González L, Laserna-Mendieta EJ, Ruiz-Ponce M, Lucendo AJ. Systematic review with meta-analysis: the growing incidence and prevalence of eosinophilic oesophagitis in children and adults in population-based studies. Aliment Pharmacol Ther. 2019 May;49(9):1116-1125. doi: 10.1111/apt.15231. Epub 2019 Mar 18. PMID: 30887555.
15. Muir A, Falk GW. Eosinophilic Esophagitis: A Review. JAMA. 2021 Oct 5;326(13):1310-1318. doi: 10.1001/jama.2021.14920. PMID: 34609446; PMCID: PMC9045493.
16. Shaheen NJ, Mukkada V, Eichinger CS, Schofield H, Todorova L, Falk GW. Natural history of eosinophilic esophagitis: a systematic review of epidemiology and disease course. Dis Esophagus. 2018 Aug 1;31(8):doy015. doi: 10.1093/dote/doy015. PMID: 29617744; PMCID: PMC6102800.
17. Straumann A. The natural history and complications of eosinophilic esophagitis. Thorac Surg Clin. 2011 Nov;21(4):575-87. doi: 10.1016/j.thorsurg.2011.09.004. PMID: 22040638.
18. Gonsalves NP, Aceves SS. Diagnosis and treatment of eosinophilic esophagitis. J Allergy Clin Immunol. 2020 Jan;145(1):1-7. doi: 10.1016/j.jaci.2019.11.011. PMID: 31910983; PMCID: PMC6986782.
19. Masrori P, Van Damme P. Amyotrophic lateral sclerosis: a clinical review. Eur J Neurol. 2020 Oct;27(10):1918-1929. doi: 10.1111/ene.14393. Epub 2020 Jul 7. PMID: 32526057; PMCID: PMC7540334.
20. Thibaut A, Beaudart C, Quinet M, Bouquiaux O, Delstanche S, Lievens I, Grosjean D, Ortmans I, Kaux JF, Halleux C. Prise en charge psychologique et cognitive au cours de l’accompagnement de fin de vie de patients atteints de sclérose latérale amyotrophique. Une revue systématique [Psychological and cognitive interventions in end-of-life support of patients with amyotrophic lateral sclerosis. A review.]. Rev Med Liege. 2022 Feb;77(2):104-109. French. PMID: 35143130.
21. van Es MA, Hardiman O, Chio A, Al-Chalabi A, Pasterkamp RJ, Veldink JH, van den Berg LH. Amyotrophic lateral sclerosis. Lancet. 2017 Nov 4;390(10107):2084-2098. doi: 10.1016/S0140-6736(17)31287-4. Epub 2017 May 25. PMID: 28552366.
